# Monopotassium monosodium hexa­hydrogen α-hexa­molybdoplatinate(IV) undeca­hydrate

**DOI:** 10.1107/S160053681000228X

**Published:** 2010-01-23

**Authors:** Uk Lee, Hea-Chung Joo

**Affiliations:** a Department of Chemistry, Pukyong National University, 599-1 Daeyeon 3-dong, Nam-gu, Busan 608-737, Republic of Korea; b Department of Chemistry, Dongeui University, San 24 Kaya-dong Busanjin-gu, Busan 614-714, Republic of Korea

## Abstract

The title compound, KNa[H_6_PtMo_6_O_24_]·11H_2_O, contains a discrete hexa­molybdoplatinate(IV) [H_6_PtMo_6_O_24_]^2−^ poly­anion  (1 symmetry), which has the highest level of protonation. Five O atoms of the central PtO_6_ octa­hedron (μ_3_-atoms, O*c*) and one O atom of an outer edge-sharing MoO_6_ octa­hedron (O bridging μ_2_-atom, O*b*) are protonated. The polyanions are connected by almost linear O—H⋯O hydrogen bonds between protonated and unprotonated O*b* atoms. Further consolidation of the crystal structure is accomplished by extensive O—H⋯O hydrogen bonding involving the uncoordinated water mol­ecules. The two independent K^+^ cations are equally disordered about a twofold rotation axis.

## Related literature

For other crystal structures containing the [H_6_PtMo_6_O_24_]^6−^ anion, see: Lee & Sasaki (1994 ▶); Lee & Joo (2006*a* ▶,*b* ▶). For background to the bond-valence method, see: Brown & Altermatt (1985 ▶); Brese & O’Keeffe (1991 ▶).
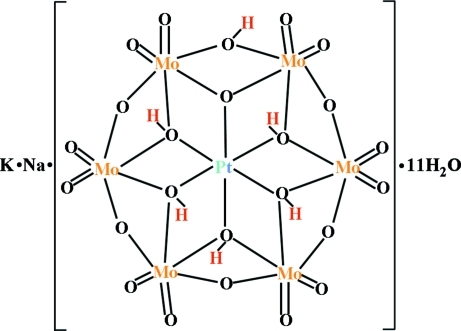


## Experimental

### 

#### Crystal data

KNa[H_6_PtMo_6_O_24_]·11H_2_O
*M* *_r_* = 1421.04
Monoclinic, 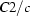

*a* = 20.935 (2) Å
*b* = 18.535 (3) Å
*c* = 17.775 (3) Å
β = 114.30 (2)°
*V* = 6286.2 (18) Å^3^
*Z* = 8
Mo *K*α radiation
μ = 7.02 mm^−1^
*T* = 298 K
0.38 × 0.25 × 0.25 mm

#### Data collection

Stoe Stadi-4 diffractometer
Absorption correction: numerical (*X-SHAPE*; Stoe & Cie 1996 ▶) *T*_min_ = 0.300, *T*_max_ = 0.422
8235 measured reflections
7237 independent reflections
5972 reflections with *I* > 2σ(*I*)
*R*_int_ = 0.028
3 standard reflections every 60 min  intensity decay: 3.2%

#### Refinement

*R*[*F* ^2^ > 2σ(*F* ^2^)] = 0.035
*wR*(*F* ^2^) = 0.082
*S* = 1.14
7237 reflections
490 parameters
33 restraints
H atoms treated by a mixture of independent and constrained refinement
Δρ_max_ = 0.96 e Å^−3^
Δρ_min_ = −1.26 e Å^−3^

### 

Data collection: *STADI4* (Stoe & Cie, 1996 ▶); cell refinement: *STADI4*; data reduction: *X-RED* (Stoe & Cie, 1996 ▶); program(s) used to solve structure: *SHELXS97* (Sheldrick, 2008 ▶); program(s) used to refine structure: *SHELXL97* (Sheldrick, 2008 ▶); molecular graphics: *ORTEP-3* (Farrugia, 1997 ▶) and *DIAMOND* (Brandenburg, 1998 ▶); software used to prepare material for publication: *SHELXL97*.

## Supplementary Material

Crystal structure: contains datablocks global, I. DOI: 10.1107/S160053681000228X/wm2298sup1.cif

Structure factors: contains datablocks I. DOI: 10.1107/S160053681000228X/wm2298Isup2.hkl

Additional supplementary materials:  crystallographic information; 3D view; checkCIF report

## Figures and Tables

**Table:** 

Pt—O1*C*	1.989 (4)
Pt—O2*C*	1.978 (4)
Pt—O3*C*	1.993 (4)
Pt—O4*C*	2.003 (4)
Pt—O5*C*	2.034 (4)
Pt—O6*C*	2.001 (4)
Mo1—O1*C*	2.327 (5)
Mo1—O6*C*	2.306 (5)
Mo1—O7*B*	1.954 (5)
Mo1—O12*B*	1.927 (5)
Mo2—O1*C*	2.317 (5)
Mo2—O2*C*	2.154 (4)
Mo2—O7*B*	1.894 (5)
Mo2—O8*B*	2.060 (5)
Mo3—O2*C*	2.163 (4)
Mo3—O3*C*	2.338 (4)
Mo3—O8*B*	2.047 (5)
Mo3—O9*B*	1.889 (5)
Mo4—O3*C*	2.323 (4)
Mo4—O4*C*	2.291 (4)
Mo4—O9*B*	1.979 (5)
Mo4—O10*B*	1.921 (5)
Mo5—O4*C*	2.328 (5)
Mo5—O5*C*	2.290 (5)
Mo5—O10*B*	1.935 (5)
Mo5—O11*B*	1.956 (4)
Mo6—O5*C*	2.302 (4)
Mo6—O6*C*	2.326 (5)
Mo6—O11*B*	1.946 (5)
Mo6—O12*B*	1.949 (5)

**Table:** 

Mo2—O1*C*—Mo1	92.06 (17)
Mo2—O2*C*—Mo3	103.72 (16)
Mo4—O3*C*—Mo3	92.16 (16)
Mo4—O4*C*—Mo5	92.48 (16)
Mo5—O5*C*—Mo6	94.18 (16)
Mo1—O6*C*—Mo6	93.33 (17)
Mo2—O7*B*—Mo1	120.6 (2)
Mo3—O8*B*—Mo2	111.6 (2)
Mo3—O9*B*—Mo4	120.5 (2)
Mo4—O10*B*—Mo5	119.8 (2)
Mo6—O11*B*—Mo5	119.1 (2)
Mo1—O12*B*—Mo6	120.7 (2)

**Table 2:** Hydrogen-bond geometry (Å, °)

*D*—H⋯*A*	*D*—H	H⋯*A*	*D*⋯*A*	*D*—H⋯*A*
O1*C*—H1⋯O10*W*^i^	0.74 (7)	1.89 (7)	2.620 (7)	174 (8)
O3*C*—H3⋯O7*W*	0.91 (8)	1.66 (8)	2.547 (7)	163 (7)
O4*C*—H4⋯O8*W*	0.79 (7)	1.82 (8)	2.594 (8)	166 (7)
O5*C*—H5⋯O9*W*	0.97 (6)	1.60 (6)	2.551 (8)	165 (7)
O6*C*—H6⋯O5*W*^ii^	0.83 (8)	1.75 (9)	2.576 (8)	179 (9)
O8*B*—H8⋯O11*B*^iii^	0.80 (7)	1.85 (7)	2.648 (6)	175 (7)
O1*W*—H1*A*⋯O2*C*^ii^	0.81 (8)	2.12 (8)	2.909 (8)	166 (13)
O1*W*—H1*B*⋯O9*B*^iv^	0.79 (8)	2.13 (9)	2.838 (8)	150 (14)
O2*W*—H2*B*⋯O24*T*	0.80 (8)	2.54 (14)	2.978 (9)	116 (13)
O3*W*—H3*A*⋯O18*T*^iv^	0.88 (8)	2.54 (14)	2.975 (10)	111 (11)
O4*W*—H4*B*⋯O20*T*^v^	0.85 (10)	2.3 (2)	2.792 (12)	113 (19)
O4*W*—H4*A*⋯O24*T*	0.82 (10)	2.39 (19)	2.957 (12)	127 (20)
O5*W*—H5*B*⋯O8*W*^ii^	0.96	2.08	2.958 (13)	151
O5*W*—H5*A*⋯O15*T*^vi^	0.96	2.01	2.687 (8)	126
O6*W*—H6*B*⋯O19*T*^iv^	0.88 (8)	2.09 (9)	2.921 (9)	158 (12)
O6*W*—H6*A*⋯O4*W*	0.98 (8)	1.90 (10)	2.788 (15)	148 (12)
O7*W*—H7*B*⋯O11*W*	0.75 (7)	2.03 (7)	2.730 (9)	155 (9)
O7*W*—H7*A*⋯O21*T*^iii^	0.96 (7)	1.88 (7)	2.727 (7)	146 (7)
O8*W*—H8*B*⋯O10*W*^vii^	0.80 (7)	2.05 (8)	2.840 (8)	167 (12)
O8*W*—H8*A*⋯O17*T*^viii^	0.76 (7)	2.27 (10)	2.867 (9)	136 (12)
O9*W*—H9*B*⋯O12*B*^i^	0.73 (8)	2.09 (10)	2.752 (8)	150 (14)
O9*W*—H9*A*⋯O6*W*^i^	0.75 (8)	2.32 (11)	2.937 (10)	141 (13)
O10*W*—H10*B*⋯O7*W*^i^	0.90 (7)	1.99 (7)	2.835 (8)	155 (9)
O10*W*—H10*A*⋯O23*T*	0.85 (7)	2.02 (7)	2.782 (8)	149 (9)
O11*W*—H11*B*⋯O10*B*	0.77 (8)	2.18 (9)	2.880 (8)	153 (14)
O11*W*—H11*A*⋯O18*T*^ix^	0.84 (7)	2.18 (8)	2.983 (8)	159 (12)

Symmetry codes: (i) 
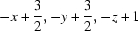
; (ii) 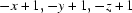; (iii) 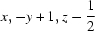; (iv) 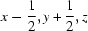; (v) 
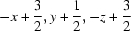
; (vi) 
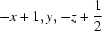
; (vii) 
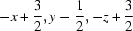
; (viii) 
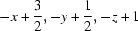
; (ix) 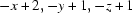.

**Fig. 1.**
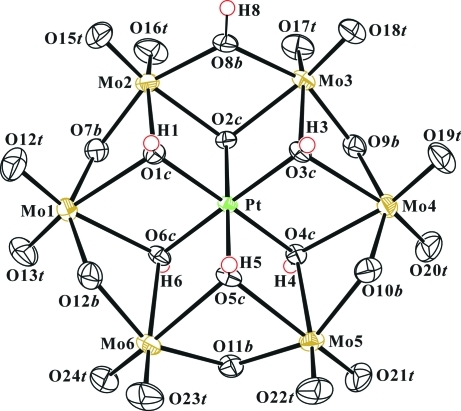
The structure of the polyanion in compound (I). Displacement ellipsoids are drawn at the 50% probability level for all non-H atoms. The latter are displayed as spheres of arbitrary radius.

**Fig. 2.**
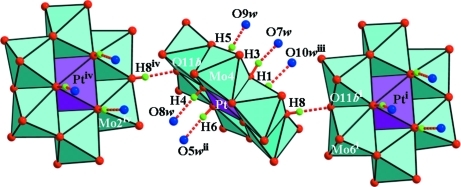
Polyhedral view of the Ob–H···Ob contact of the inter-anion hydrogen bond and Oc–H···Owhydrogen bonds shown as dashed lines. [Symmetry codes: (i) x, - y + 1, z - 1/2; (ii) - x + 1, - y + 1, - z + 1; (iii) - x + 3/2, - y + 3/2, - z + 1; (vi) x, - y + 1, z + 1/2.]

## supplementary crystallographic information

### Comment

In our previous studies we isolated the same polyanion, [H_6_PtMo_6_O_24_]^2-^, that is present in the title compound, (I), at various pH conditions; 1.60 (II; Lee & Joo, 2006*b*), 0.70 (III; Lee & Sasaki, 1994) and 0.48 (IV; Lee & Joo, 2006*a*). Structures (II, III) have the same space group, *viz*. *C*2/*c*, whereas the space group of (IV) is *P*1. The polyanions bear an inversion center in these three structures. The current study was carried out to confirm the presence of a highly protonated species that exists at very low pH.

The structure of the present crystals contains a crystallographically discrete [H_6_PMo_6_O_24_]^2-^ polyanion (Fig. 1). All atoms in the polyanion are located in general positions and consequently the symmetry of the polyanion is *C*1. The O atoms of the polyoxometalate were designated as O*t* (terminal Mo═O atom), O*b* (O bridging µ_2_-O atom), and O*c* (µ_3_-O atom). The protonated O atoms in the polyanion were identified in difference Fourier maps and by using structural features as observed in the previously determined structures II & IV, *viz*. bond lengths of Mo–O*c*(H) & Mo–O*b*(H) units, bond angles of Mo–O*c*(H)–Mo & Mo–O*b*(H)–Mo units, and distances between Mo···Mo. As a result, the structure of (I) confirms the protonation of atoms O1*c*(H), O3*c*(H), O4*c*(H), O5*c*(H), O6*c*(H) and O8*b*(H).

The different bond-lengths and bond-angles in the [H_6_PtMo_6_O_24_]^2-^ polyanion of protonated and unprotonated O atoms are compared in Table 1. The protonated O atoms of [H_6_PtMo_6_O_24_]^2-^ in the structures (II), (III) and (IV) show the same protonation scheme, *viz*. four O*c*(H) and two O*b*(H) atoms are protonated. Therefore, the feature of the three-dimensional hydrogen bonding formation is very similar in the these polyanions, *viz*. the central PtO_2_(OH)_4_ polyhedron forms hydrogen bonds with neighbouring polyanions by four sets of O*c*(H)···O*t* and O*b*(H)···O*t* hydrogen bonds.

However, the protonation scheme of the polyanion in (I) is different, consisting of five O*c*(H) and one O*b*(H) protonated O atoms (Fig. 1). In contrast to the hydrogen bonding scheme in (II-IV), the protonated O*c* atoms form various O–H···O hydrogen bonds with water molecules (O*w*). Nevertheless, the polyanion is linearly connected by an O8*b*–H8···O11*b*^i^ hydrogen bond (Fig.2 and Table 2).

The Na and K ions are coordinated by O atoms as [Na(O*w*)_5_(O*t*)]^+^ in the range 2.371 (7)-2.510 (9) Å, and [K1(O*t*)_4_(O*w*)_2_]^+^ and [K2(O*t*)_3_(O*w*)_4_]^+^ in the range 2.59 (1)-3.41 (1) Å. Bond valence sum calculations (BVS; Brown & Altermatt, 1985; Brese & O'Keeffe, 1991) for the K1 and K2 ions reveal a considerable undersaturation in terms of valence units which we ascribe to the disordered character of the metal positions.

### Experimental

Crystals of the title compound were prepared by the reaction of Na_2_MoO_4_.2H_2_O and K_2_Pt(OH)_6_ at pH 0.20 as described in a previous report (Lee & Sasaki, 1994).

### Refinement

The O*c* and O*b* bound H atoms in the polyanion were located in difference Fourier maps and were freely refined except H5. H5 was refined with a distance restraint [O5*c*–H5 = 0.85 (10) Å]. All water molecules bound H atoms were located in difference Fourier maps and their positional parameters were refined with a distance restraint [O–H = 0.85 (10) Å, but O6*w*–6HA = 0.75 (10) Å] and an additional angle restraint; these H atoms were refined with an isotropic displacement parameter *U*_iso_ = 1.5*U*_eq_(O). O5*w* bound H atoms were placed in calculated positions. They were included in the refinement using the riding-motion approximation, with *U*_iso_(H) = 1.5 *U*_eq_(O). K1 and K2 showed very large displacement parameters under consideration of full occupation. Refinement of the site occupation factors (s.o.f.) converged at values close to half-occupation. In the final refinement the s.o.f.'s were constrained to 0.5 and reasonable displacement parameters were eventually obtained.

### Crystal data

**Table:**

KNa[H_6_PtMo_6_O_24_]·11H_2_O	*F*(000) = 5344
*M**_r_* = 1421.04	*D*_x_ = 3.003 Mg m^−^^3^
Monoclinic, *C*2/*c*	Mo *K*α radiation, λ = 0.71069 Å
Hall symbol: -C 2yc	Cell parameters from 25 reflections
*a* = 20.935 (2) Å	θ = 9.6–10.3°
*b* = 18.535 (3) Å	µ = 7.02 mm^−^^1^
*c* = 17.775 (3) Å	*T* = 298 K
β = 114.30 (2)°	Block, pale yellow
*V* = 6286.2 (18) Å^3^	0.38 × 0.25 × 0.25 mm
*Z* = 8	

### Data collection

**Table:**

Stoe Stadi-4 diffractometer	5972 reflections with *I* > 2σ(*I*)
Radiation source: fine-focus sealed tube	*R*_int_ = 0.028
graphite	θ_max_ = 27.5°, θ_min_ = 1.5°
ω/2–θ scans	*h* = −2→27
Absorption correction: numerical (*X-SHAPE*; Stoe & Cie 1996)	*k* = −24→24
*T*_min_ = 0.300, *T*_max_ = 0.422	*l* = −23→21
8235 measured reflections	3 standard reflections every 60 min
7237 independent reflections	intensity decay: 3.2%

### Refinement

**Table:**

Refinement on *F*^2^	Primary atom site location: structure-invariant direct methods
Least-squares matrix: full	Secondary atom site location: difference Fourier map
*R*[*F*^2^ > 2σ(*F*^2^)] = 0.035	Hydrogen site location: difference Fourier map
*wR*(*F*^2^) = 0.082	H atoms treated by a mixture of independent and constrained refinement
*S* = 1.14	*w* = 1/[σ^2^(*F*_o_^2^) + (0.0282*P*)^2^ + 43.9139*P*] where *P* = (*F*_o_^2^ + 2*F*_c_^2^)/3
7237 reflections	(Δ/σ)_max_ = 0.001
490 parameters	Δρ_max_ = 0.96 e Å^−^^3^
33 restraints	Δρ_min_ = −1.26 e Å^−^^3^

### Special details

**Table:**

Geometry. All e.s.d.'s (except the e.s.d. in the dihedral angle between two l.s. planes) are estimated using the full covariance matrix. The cell e.s.d.'s are taken into account individually in the estimation of e.s.d.'s in distances, angles and torsion angles; correlations between e.s.d.'s in cell parameters are only used when they are defined by crystal symmetry. An approximate (isotropic) treatment of cell e.s.d.'s is used for estimating e.s.d.'s involving l.s. planes.
Refinement. Refinement of *F*^2^ against ALL reflections. The weighted *R*-factor *wR* and goodness of fit *S* are based on *F*^2^, conventional *R*-factors *R* are based on *F*, with *F* set to zero for negative *F*^2^. The threshold expression of *F*^2^ > σ(*F*^2^) is used only for calculating *R*-factors(gt) *etc*. and is not relevant to the choice of reflections for refinement. *R*-factors based on *F*^2^ are statistically about twice as large as those based on *F*, and *R*- factors based on ALL data will be even larger.

### Fractional atomic coordinates and isotropic or equivalent isotropic displacement parameters (Å^2^)

**Table:**

	*x*	*y*	*z*	*U*_iso_*/*U*_eq_	Occ. (<1)
Pt	0.755879 (11)	0.504087 (12)	0.501298 (14)	0.01487 (6)	
Mo1	0.60877 (3)	0.60494 (3)	0.40899 (4)	0.02644 (13)	
Mo2	0.64099 (3)	0.46279 (3)	0.31418 (3)	0.02522 (13)	
Mo3	0.78857 (3)	0.36281 (3)	0.40922 (4)	0.02288 (12)	
Mo4	0.90273 (3)	0.40566 (3)	0.60119 (4)	0.02459 (13)	
Mo5	0.86645 (3)	0.54226 (3)	0.69946 (3)	0.02323 (12)	
Mo6	0.71872 (3)	0.63908 (3)	0.60625 (4)	0.02426 (13)	
K1	0.4929 (4)	0.7725 (2)	0.2195 (4)	0.092 (4)	0.50
K2	0.5123 (4)	0.7131 (4)	0.7936 (5)	0.114 (3)	0.50
Na	0.45373 (19)	0.66261 (18)	0.4760 (2)	0.0478 (8)	
O1C	0.7062 (2)	0.5568 (2)	0.3956 (3)	0.0209 (9)	
H1	0.726 (4)	0.583 (4)	0.381 (5)	0.02 (2)*	
O2C	0.7085 (2)	0.4197 (2)	0.4330 (3)	0.0194 (9)	
O3C	0.8374 (2)	0.4689 (2)	0.4811 (3)	0.0189 (9)	
H3	0.866 (4)	0.501 (4)	0.471 (5)	0.03 (2)*	
O4C	0.8038 (2)	0.4515 (2)	0.6085 (3)	0.0185 (9)	
H4	0.781 (4)	0.429 (4)	0.626 (4)	0.022 (19)*	
O5C	0.8043 (2)	0.5910 (2)	0.5715 (3)	0.0212 (9)	
H5	0.830 (3)	0.624 (4)	0.551 (4)	0.025 (19)*	
O6C	0.6747 (2)	0.5377 (2)	0.5240 (3)	0.0215 (9)	
H6	0.654 (4)	0.509 (5)	0.542 (5)	0.04 (2)*	
O7B	0.5858 (2)	0.5080 (3)	0.3625 (3)	0.0286 (10)	
O8B	0.7364 (2)	0.4336 (3)	0.3155 (3)	0.0265 (10)	
H8	0.747 (3)	0.435 (4)	0.277 (4)	0.016 (17)*	
O9B	0.8324 (2)	0.3398 (2)	0.5227 (3)	0.0264 (10)	
O10B	0.9231 (2)	0.5009 (3)	0.6477 (3)	0.0279 (10)	
O11B	0.7729 (2)	0.5689 (3)	0.6895 (3)	0.0258 (10)	
O12B	0.6770 (3)	0.6662 (3)	0.4900 (3)	0.0310 (11)	
O13T	0.5397 (3)	0.6145 (3)	0.4351 (4)	0.0463 (15)	
O14T	0.5911 (3)	0.6567 (3)	0.3234 (3)	0.0448 (14)	
O15T	0.6253 (3)	0.5154 (4)	0.2295 (3)	0.0504 (16)	
O16T	0.5949 (3)	0.3850 (3)	0.2793 (4)	0.0476 (15)	
O17T	0.7403 (3)	0.2868 (3)	0.3706 (4)	0.0409 (13)	
O18T	0.8564 (3)	0.3585 (3)	0.3792 (3)	0.0365 (12)	
O19T	0.9696 (3)	0.3948 (3)	0.5710 (4)	0.0408 (13)	
O20T	0.9220 (3)	0.3524 (3)	0.6861 (3)	0.0433 (14)	
O21T	0.8880 (3)	0.4880 (3)	0.7839 (3)	0.0403 (13)	
O22T	0.9118 (3)	0.6211 (3)	0.7340 (3)	0.0398 (13)	
O23T	0.7642 (3)	0.7167 (3)	0.6445 (4)	0.0423 (14)	
O24T	0.6480 (3)	0.6418 (3)	0.6303 (3)	0.0419 (14)	
O1W	0.3734 (4)	0.6928 (4)	0.5338 (6)	0.072 (2)	
H1A	0.357 (7)	0.658 (5)	0.547 (8)	0.108*	
H1B	0.349 (6)	0.727 (5)	0.518 (9)	0.108*	
O2W	0.5059 (4)	0.5835 (6)	0.5945 (5)	0.089 (3)	
H2A	0.478 (6)	0.607 (9)	0.604 (9)	0.133*	
H2B	0.541 (5)	0.582 (8)	0.636 (7)	0.133*	
O3W	0.4069 (5)	0.7211 (5)	0.3363 (5)	0.075 (2)	
H3A	0.394 (7)	0.761 (5)	0.306 (8)	0.113*	
H3B	0.380 (7)	0.692 (6)	0.309 (8)	0.113*	
O4W	0.6004 (9)	0.7658 (7)	0.6985 (7)	0.151 (6)	
H4A	0.624 (11)	0.729 (7)	0.714 (14)	0.227*	
H4B	0.624 (11)	0.802 (8)	0.726 (13)	0.227*	
O5W	0.3899 (4)	0.5535 (5)	0.4228 (4)	0.085 (3)	
H5A	0.4118	0.5270	0.3935	0.128*	
H5B	0.3427	0.5653	0.3859	0.128*	
O6W	0.5264 (4)	0.7653 (4)	0.5274 (6)	0.071 (2)	
H6A	0.538 (7)	0.755 (7)	0.586 (5)	0.106*	
H6B	0.501 (6)	0.803 (5)	0.526 (8)	0.106*	
O7W	0.8968 (3)	0.5657 (3)	0.4306 (3)	0.0335 (12)	
H7A	0.909 (4)	0.560 (5)	0.385 (4)	0.050*	
H7B	0.929 (4)	0.582 (5)	0.465 (5)	0.050*	
O8W	0.7388 (4)	0.3605 (4)	0.6632 (4)	0.0536 (18)	
H8A	0.757 (6)	0.335 (6)	0.645 (7)	0.080*	
H8B	0.749 (6)	0.367 (6)	0.711 (5)	0.080*	
O9W	0.8666 (4)	0.6922 (3)	0.5311 (6)	0.068 (2)	
H9A	0.904 (4)	0.694 (7)	0.537 (8)	0.102*	
H9B	0.844 (6)	0.723 (6)	0.529 (9)	0.102*	
O10W	0.7273 (3)	0.8572 (3)	0.6648 (3)	0.0384 (13)	
H10A	0.729 (5)	0.819 (4)	0.639 (6)	0.058*	
H10B	0.682 (4)	0.870 (5)	0.639 (6)	0.058*	
O11W	0.9966 (3)	0.5975 (5)	0.5839 (4)	0.065 (2)	
H11A	1.040 (4)	0.599 (7)	0.601 (7)	0.098*	
H11B	0.982 (6)	0.581 (7)	0.613 (7)	0.098*	

### Atomic displacement parameters (Å^2^)

**Table:**

	*U*^11^	*U*^22^	*U*^33^	*U*^12^	*U*^13^	*U*^23^
Pt	0.01848 (11)	0.01434 (11)	0.01480 (10)	−0.00019 (9)	0.00989 (8)	−0.00024 (8)
Mo1	0.0277 (3)	0.0272 (3)	0.0253 (3)	0.0088 (2)	0.0118 (2)	0.0026 (2)
Mo2	0.0240 (3)	0.0335 (3)	0.0178 (3)	0.0001 (2)	0.0084 (2)	−0.0043 (2)
Mo3	0.0284 (3)	0.0189 (3)	0.0263 (3)	0.0011 (2)	0.0163 (2)	−0.0031 (2)
Mo4	0.0230 (3)	0.0280 (3)	0.0255 (3)	0.0061 (2)	0.0128 (2)	0.0037 (2)
Mo5	0.0232 (3)	0.0303 (3)	0.0176 (3)	−0.0037 (2)	0.0098 (2)	−0.0032 (2)
Mo6	0.0325 (3)	0.0199 (3)	0.0252 (3)	0.0021 (2)	0.0168 (2)	−0.0031 (2)
K1	0.041 (4)	0.039 (2)	0.183 (10)	−0.008 (2)	0.035 (6)	−0.025 (3)
K2	0.079 (4)	0.102 (5)	0.174 (8)	−0.040 (4)	0.067 (6)	−0.056 (5)
Na	0.055 (2)	0.0396 (18)	0.064 (2)	−0.0055 (16)	0.0407 (18)	−0.0080 (17)
O1C	0.026 (2)	0.020 (2)	0.019 (2)	0.0016 (19)	0.0117 (19)	0.0037 (18)
O2C	0.023 (2)	0.020 (2)	0.016 (2)	−0.0006 (17)	0.0089 (17)	−0.0009 (17)
O3C	0.021 (2)	0.018 (2)	0.024 (2)	−0.0002 (17)	0.0147 (18)	−0.0002 (18)
O4C	0.023 (2)	0.020 (2)	0.019 (2)	0.0016 (18)	0.0142 (18)	0.0068 (18)
O5C	0.027 (2)	0.018 (2)	0.024 (2)	−0.0026 (18)	0.0154 (19)	−0.0044 (18)
O6C	0.026 (2)	0.021 (2)	0.025 (2)	0.0018 (19)	0.0172 (19)	−0.0008 (19)
O7B	0.022 (2)	0.037 (3)	0.027 (2)	0.000 (2)	0.0098 (19)	−0.003 (2)
O8B	0.029 (2)	0.036 (3)	0.020 (2)	0.002 (2)	0.015 (2)	−0.002 (2)
O9B	0.035 (3)	0.017 (2)	0.029 (2)	0.0049 (19)	0.016 (2)	0.0032 (19)
O10B	0.020 (2)	0.038 (3)	0.026 (2)	−0.009 (2)	0.0107 (18)	−0.005 (2)
O11B	0.032 (2)	0.030 (3)	0.022 (2)	0.002 (2)	0.017 (2)	0.0037 (19)
O12B	0.041 (3)	0.022 (2)	0.032 (3)	0.007 (2)	0.017 (2)	0.006 (2)
O13T	0.039 (3)	0.054 (4)	0.052 (4)	0.012 (3)	0.025 (3)	−0.008 (3)
O14T	0.048 (3)	0.042 (3)	0.035 (3)	0.010 (3)	0.008 (3)	0.012 (3)
O15T	0.047 (3)	0.081 (5)	0.025 (3)	0.026 (3)	0.017 (3)	0.015 (3)
O16T	0.034 (3)	0.059 (4)	0.048 (3)	−0.015 (3)	0.016 (3)	−0.029 (3)
O17T	0.055 (4)	0.024 (3)	0.047 (3)	−0.008 (2)	0.024 (3)	−0.012 (2)
O18T	0.038 (3)	0.042 (3)	0.036 (3)	0.008 (2)	0.022 (2)	−0.001 (2)
O19T	0.030 (3)	0.047 (3)	0.055 (3)	0.006 (2)	0.027 (3)	−0.005 (3)
O20T	0.044 (3)	0.049 (4)	0.031 (3)	0.011 (3)	0.010 (2)	0.015 (3)
O21T	0.038 (3)	0.060 (4)	0.023 (2)	0.010 (3)	0.013 (2)	0.012 (3)
O22T	0.038 (3)	0.044 (3)	0.037 (3)	−0.018 (3)	0.015 (2)	−0.016 (3)
O23T	0.064 (4)	0.020 (3)	0.046 (3)	−0.008 (3)	0.025 (3)	−0.009 (2)
O24T	0.042 (3)	0.054 (4)	0.037 (3)	0.012 (3)	0.025 (3)	−0.001 (3)
O1W	0.085 (5)	0.031 (4)	0.139 (7)	0.015 (4)	0.086 (6)	0.021 (4)
O2W	0.040 (4)	0.134 (9)	0.067 (5)	−0.022 (5)	−0.005 (4)	0.044 (6)
O3W	0.091 (6)	0.064 (5)	0.063 (5)	0.017 (5)	0.025 (5)	0.004 (4)
O4W	0.278 (19)	0.086 (8)	0.088 (8)	0.070 (10)	0.074 (10)	−0.009 (6)
O5W	0.113 (6)	0.110 (7)	0.058 (4)	−0.082 (5)	0.062 (5)	−0.047 (4)
O6W	0.066 (5)	0.045 (4)	0.106 (6)	0.000 (4)	0.041 (5)	−0.011 (4)
O7W	0.033 (3)	0.040 (3)	0.034 (3)	−0.012 (2)	0.020 (2)	−0.003 (2)
O8W	0.077 (5)	0.052 (4)	0.047 (4)	−0.028 (4)	0.041 (4)	−0.012 (3)
O9W	0.074 (5)	0.030 (3)	0.140 (7)	0.012 (3)	0.084 (6)	0.022 (4)
O10W	0.049 (3)	0.034 (3)	0.037 (3)	−0.002 (3)	0.024 (3)	−0.003 (2)
O11W	0.033 (3)	0.113 (7)	0.042 (4)	−0.021 (4)	0.006 (3)	0.020 (4)

### Geometric parameters (Å, °)

**Table:**

Mo1—Mo2	3.3426 (9)	Mo4—O20T	1.707 (5)
Mo1—Mo6	3.3690 (12)	Mo5—O4C	2.328 (5)
Mo2—Mo3	3.3968 (10)	Mo5—O5C	2.290 (5)
Mo3—Mo4	3.3577 (12)	Mo5—O10B	1.935 (5)
Mo4—Mo5	3.3363 (9)	Mo5—O11B	1.956 (4)
Mo5—Mo6	3.3635 (10)	Mo5—O21T	1.705 (5)
Pt—O1C	1.989 (4)	Mo5—O22T	1.713 (5)
Pt—O2C	1.978 (4)	Mo6—O5C	2.302 (4)
Pt—O3C	1.993 (4)	Mo6—O6C	2.326 (5)
Pt—O4C	2.003 (4)	Mo6—O11B	1.946 (5)
Pt—O5C	2.034 (4)	Mo6—O12B	1.949 (5)
Pt—O6C	2.001 (4)	Mo6—O23T	1.704 (5)
Mo1—O1C	2.327 (5)	Mo6—O24T	1.702 (5)
Mo1—O6C	2.306 (5)	K1—O14T^i^	2.678 (8)
Mo1—O7B	1.954 (5)	K1—O14T	3.020 (8)
Mo1—O12B	1.927 (5)	K1—O22T^ii^	2.682 (9)
Mo1—O13T	1.701 (5)	K1—O22T^iii^	2.683 (8)
Mo1—O14T	1.706 (5)	K1—O9W^ii^	3.368 (12)
Mo2—O1C	2.317 (5)	K1—O3W	3.396 (14)
Mo2—O2C	2.154 (4)	K1—O3W^i^	2.831 (14)
Mo2—O7B	1.894 (5)	K2—O16T^iv^	2.594 (10)
Mo2—O8B	2.060 (5)	K2—O16T^v^	2.763 (8)
Mo2—O15T	1.708 (6)	K2—O20T^vi^	2.878 (9)
Mo2—O16T	1.703 (6)	K2—O20T^vii^	3.305 (10)
Mo3—O2C	2.163 (4)	K2—O1W^viii^	3.039 (13)
Mo3—O3C	2.338 (4)	K2—O2W^viii^	3.239 (14)
Mo3—O8B	2.047 (5)	K2—O4W	3.13 (2)
Mo3—O9B	1.889 (5)	K2—O4W^viii^	2.61 (2)
Mo3—O17T	1.705 (5)	Na—O13T	2.374 (6)
Mo3—O18T	1.712 (5)	Na—O1W	2.371 (7)
Mo4—O3C	2.323 (4)	Na—O2W	2.425 (9)
Mo4—O4C	2.291 (4)	Na—O3W	2.510 (9)
Mo4—O9B	1.979 (5)	Na—O5W	2.393 (7)
Mo4—O10B	1.921 (5)	Na—O6W	2.373 (9)
Mo4—O19T	1.706 (5)		
			
Mo2—Mo1—Mo6	118.66 (2)	O12B—Mo6—O5C	82.81 (18)
Mo1—Mo2—Mo3	120.68 (3)	O24T—Mo6—O6C	91.2 (2)
Mo4—Mo3—Mo2	119.85 (3)	O23T—Mo6—O6C	160.8 (2)
Mo5—Mo4—Mo3	119.43 (2)	O11B—Mo6—O6C	83.89 (18)
Mo4—Mo5—Mo6	120.55 (3)	O12B—Mo6—O6C	69.74 (18)
Mo5—Mo6—Mo1	120.55 (3)	O5C—Mo6—O6C	70.70 (15)
O2C—Pt—O1C	82.75 (18)	Pt—O1C—Mo2	99.52 (19)
O2C—Pt—O3C	83.14 (17)	Pt—O1C—Mo1	103.37 (19)
O1C—Pt—O3C	98.28 (18)	Pt—O2C—Mo2	105.61 (19)
O2C—Pt—O6C	96.73 (18)	Pt—O2C—Mo3	105.81 (18)
O1C—Pt—O6C	83.14 (19)	Pt—O3C—Mo4	102.91 (17)
O3C—Pt—O6C	178.53 (18)	Pt—O3C—Mo3	99.17 (17)
O2C—Pt—O4C	97.30 (17)	Pt—O4C—Mo4	103.71 (18)
O1C—Pt—O4C	178.52 (18)	Pt—O4C—Mo5	103.15 (18)
O3C—Pt—O4C	83.19 (17)	Pt—O5C—Mo5	103.48 (19)
O6C—Pt—O4C	95.39 (18)	Pt—O5C—Mo6	102.98 (18)
O2C—Pt—O5C	179.86 (18)	Pt—O6C—Mo1	103.74 (19)
O1C—Pt—O5C	97.20 (19)	Pt—O6C—Mo6	103.20 (19)
O3C—Pt—O5C	97.00 (17)	Mo2—O1C—Mo1	92.06 (17)
O6C—Pt—O5C	83.13 (18)	Mo2—O2C—Mo3	103.72 (16)
O4C—Pt—O5C	82.74 (18)	Mo4—O3C—Mo3	92.16 (16)
O13T—Mo1—O14T	106.8 (3)	Mo4—O4C—Mo5	92.48 (16)
O13T—Mo1—O12B	100.7 (3)	Mo5—O5C—Mo6	94.18 (16)
O14T—Mo1—O12B	99.0 (3)	Mo1—O6C—Mo6	93.33 (17)
O13T—Mo1—O7B	97.1 (3)	Mo2—O7B—Mo1	120.6 (2)
O14T—Mo1—O7B	102.4 (3)	Mo3—O8B—Mo2	111.6 (2)
O12B—Mo1—O7B	146.8 (2)	Mo3—O9B—Mo4	120.5 (2)
O13T—Mo1—O6C	94.6 (2)	Mo4—O10B—Mo5	119.8 (2)
O14T—Mo1—O6C	157.8 (2)	Mo6—O11B—Mo5	119.1 (2)
O12B—Mo1—O6C	70.55 (18)	Mo1—O12B—Mo6	120.7 (2)
O7B—Mo1—O6C	80.28 (18)	H1A—O1W—H1B	118 (10)
O13T—Mo1—O1C	161.4 (2)	H2A—O2W—H2B	107 (10)
O14T—Mo1—O1C	90.1 (2)	H3A—O3W—H3B	103 (10)
O12B—Mo1—O1C	83.76 (19)	H4A—O4W—H4B	110 (10)
O7B—Mo1—O1C	71.17 (18)	H5A—O5W—H5B	109.5
O6C—Mo1—O1C	69.71 (15)	H6A—O6W—H6B	93 (8)
O16T—Mo2—O15T	106.7 (3)	H7A—O7W—H7B	106 (7)
O16T—Mo2—O7B	100.3 (2)	H8A—O8W—H8B	126 (10)
O15T—Mo2—O7B	102.4 (2)	H9A—O9W—H9B	126 (10)
O16T—Mo2—O8B	100.5 (2)	H10A—O10W—H10B	103 (7)
O15T—Mo2—O8B	89.7 (2)	H11A—O11W—H11B	117 (10)
O7B—Mo2—O8B	151.67 (19)	O14T^i^—K1—O22T^ii^	104.4 (4)
O16T—Mo2—O2C	95.1 (2)	O14T^i^—K1—O22T^iii^	174.0 (4)
O15T—Mo2—O2C	152.4 (2)	O22T^ii^—K1—O22T^iii^	81.4 (3)
O7B—Mo2—O2C	89.79 (18)	O14T^i^—K1—O3W^i^	97.1 (3)
O8B—Mo2—O2C	69.47 (17)	O22T^ii^—K1—O3W^i^	150.6 (4)
O16T—Mo2—O1C	164.6 (2)	O22T^iii^—K1—O3W^i^	77.9 (3)
O15T—Mo2—O1C	88.4 (3)	O14T^i^—K1—O14T	78.8 (3)
O7B—Mo2—O1C	72.37 (18)	O22T^ii^—K1—O14T	139.7 (3)
O8B—Mo2—O1C	82.60 (18)	O22T^iii^—K1—O14T	95.8 (3)
O2C—Mo2—O1C	71.70 (16)	O3W^i^—K1—O14T	63.8 (3)
O17T—Mo3—O18T	105.4 (3)	O14T^i^—K1—O9W^ii^	73.6 (2)
O17T—Mo3—O9B	100.9 (2)	O22T^ii^—K1—O9W^ii^	71.6 (2)
O18T—Mo3—O9B	102.8 (2)	O22T^iii^—K1—O9W^ii^	110.1 (3)
O17T—Mo3—O8B	99.9 (2)	O3W^i^—K1—O9W^ii^	96.2 (3)
O18T—Mo3—O8B	90.6 (2)	O14T—K1—O9W^ii^	143.5 (2)
O9B—Mo3—O8B	151.14 (19)	O14T^i^—K1—O3W	59.9 (3)
O17T—Mo3—O2C	95.9 (2)	O22T^ii^—K1—O3W	68.4 (3)
O18T—Mo3—O2C	153.2 (2)	O22T^iii^—K1—O3W	122.2 (3)
O9B—Mo3—O2C	88.67 (18)	O3W^i^—K1—O3W	140.8 (4)
O8B—Mo3—O2C	69.52 (17)	O14T—K1—O3W	80.0 (2)
O17T—Mo3—O3C	165.5 (2)	O9W^ii^—K1—O3W	105.4 (3)
O18T—Mo3—O3C	88.7 (2)	O16T^iv^—K2—O4W^viii^	157.2 (4)
O9B—Mo3—O3C	72.29 (18)	O16T^iv^—K2—O16T^v^	86.4 (3)
O8B—Mo3—O3C	82.70 (18)	O4W^viii^—K2—O16T^v^	72.2 (3)
O2C—Mo3—O3C	71.54 (15)	O16T^iv^—K2—O20T^vi^	109.7 (3)
O19T—Mo4—O20T	106.5 (3)	O4W^viii^—K2—O20T^vi^	93.1 (3)
O19T—Mo4—O10B	100.3 (2)	O16T^v^—K2—O20T^vi^	154.7 (4)
O20T—Mo4—O10B	102.8 (3)	O16T^iv^—K2—O1W^viii^	74.5 (3)
O19T—Mo4—O9B	100.0 (2)	O4W^viii^—K2—O1W^viii^	110.2 (4)
O20T—Mo4—O9B	95.9 (2)	O16T^v^—K2—O1W^viii^	122.8 (3)
O10B—Mo4—O9B	147.10 (19)	O20T^vi^—K2—O1W^viii^	81.2 (2)
O19T—Mo4—O4C	160.1 (2)	O16T^iv^—K2—O4W	66.4 (3)
O20T—Mo4—O4C	93.1 (2)	O4W^viii^—K2—O4W	131.3 (5)
O10B—Mo4—O4C	71.40 (17)	O16T^v^—K2—O4W	119.9 (4)
O9B—Mo4—O4C	80.87 (17)	O20T^vi^—K2—O4W	55.2 (3)
O19T—Mo4—O3C	91.1 (2)	O1W^viii^—K2—O4W	100.9 (3)
O20T—Mo4—O3C	160.0 (2)	O16T^iv^—K2—O2W^viii^	78.0 (3)
O10B—Mo4—O3C	82.77 (18)	O4W^viii^—K2—O2W^viii^	84.7 (3)
O9B—Mo4—O3C	71.18 (17)	O16T^v^—K2—O2W^viii^	61.4 (2)
O4C—Mo4—O3C	70.19 (14)	O20T^vi^—K2—O2W^viii^	139.4 (3)
O21T—Mo5—O22T	106.3 (3)	O1W^viii^—K2—O2W^viii^	62.0 (2)
O21T—Mo5—O10B	101.2 (2)	O4W—K2—O2W^viii^	143.9 (4)
O22T—Mo5—O10B	98.7 (2)	O16T^iv^—K2—O20T^vii^	137.5 (3)
O21T—Mo5—O11B	96.9 (2)	O4W^viii^—K2—O20T^vii^	54.8 (3)
O22T—Mo5—O11B	101.8 (2)	O16T^v^—K2—O20T^vii^	94.7 (3)
O10B—Mo5—O11B	147.60 (19)	O20T^vi^—K2—O20T^vii^	60.1 (3)
O21T—Mo5—O5C	160.5 (2)	O1W^viii^—K2—O20T^vii^	134.9 (3)
O22T—Mo5—O5C	91.4 (2)	O4W—K2—O20T^vii^	76.6 (3)
O10B—Mo5—O5C	83.82 (18)	O2W^viii^—K2—O20T^vii^	138.6 (3)
O11B—Mo5—O5C	70.87 (17)	O1W—Na—O6W	95.8 (3)
O21T—Mo5—O4C	93.1 (2)	O1W—Na—O13T	169.6 (3)
O22T—Mo5—O4C	159.4 (2)	O6W—Na—O13T	88.4 (3)
O10B—Mo5—O4C	70.36 (17)	O1W—Na—O5W	89.5 (3)
O11B—Mo5—O4C	82.08 (18)	O6W—Na—O5W	174.4 (3)
O5C—Mo5—O4C	70.60 (15)	O13T—Na—O5W	86.1 (3)
O24T—Mo6—O23T	106.0 (3)	O1W—Na—O2W	85.0 (3)
O24T—Mo6—O11B	97.1 (2)	O6W—Na—O2W	99.4 (4)
O23T—Mo6—O11B	101.9 (2)	O13T—Na—O2W	85.0 (3)
O24T—Mo6—O12B	101.6 (2)	O5W—Na—O2W	79.1 (3)
O23T—Mo6—O12B	97.9 (2)	O1W—Na—O3W	105.6 (3)
O11B—Mo6—O12B	147.71 (19)	O6W—Na—O3W	87.8 (3)
O24T—Mo6—O5C	158.8 (2)	O13T—Na—O3W	84.0 (3)
O23T—Mo6—O5C	93.7 (2)	O5W—Na—O3W	92.7 (3)
O11B—Mo6—O5C	70.77 (17)	O2W—Na—O3W	166.7 (4)

Symmetry codes: (i) −*x*+1, *y*, −*z*+1/2; (ii) *x*−1/2, −*y*+3/2, *z*−1/2; (iii) −*x*+3/2, −*y*+3/2, −*z*+1; (iv) *x*, −*y*+1, *z*+1/2; (v) −*x*+1, −*y*+1, −*z*+1; (vi) −*x*+3/2, *y*+1/2, −*z*+3/2; (vii) *x*−1/2, *y*+1/2, *z*; (viii) −*x*+1, *y*, −*z*+3/2.

### Hydrogen-bond geometry (Å, °)

**Table:**

*D*—H···*A*	*D*—H	H···*A*	*D*···*A*	*D*—H···*A*
O1C—H1···O10W^iii^	0.74 (7)	1.89 (7)	2.620 (7)	174 (8)
O3C—H3···O7W	0.91 (8)	1.66 (8)	2.547 (7)	163 (7)
O4C—H4···O8W	0.79 (7)	1.82 (8)	2.594 (8)	166 (7)
O5C—H5···O9W	0.97 (6)	1.60 (6)	2.551 (8)	165 (7)
O6C—H6···O5W^v^	0.83 (8)	1.75 (9)	2.576 (8)	179 (9)
O8B—H8···O11B^ix^	0.80 (7)	1.85 (7)	2.648 (6)	175 (7)
O1W—H1A···O2C^v^	0.81 (8)	2.12 (8)	2.909 (8)	166 (13)
O1W—H1B···O9B^vii^	0.79 (8)	2.13 (9)	2.838 (8)	150 (14)
O2W—H2B···O24T	0.80 (8)	2.54 (14)	2.978 (9)	116 (13)
O3W—H3A···O18T^vii^	0.88 (8)	2.54 (14)	2.975 (10)	111 (11)
O4W—H4B···O20T^vi^	0.85 (10)	2.3 (2)	2.792 (12)	113 (19)
O4W—H4A···O24T	0.82 (10)	2.39 (19)	2.957 (12)	127 (20)
O5W—H5B···O8W^v^	0.96	2.08	2.958 (13)	151
O5W—H5A···O15T^i^	0.96	2.01	2.687 (8)	126
O6W—H6B···O19T^vii^	0.88 (8)	2.09 (9)	2.921 (9)	158 (12)
O6W—H6A···O4W	0.98 (8)	1.90 (10)	2.788 (15)	148 (12)
O7W—H7B···O11W	0.75 (7)	2.03 (7)	2.730 (9)	155 (9)
O7W—H7A···O21T^ix^	0.96 (7)	1.88 (7)	2.727 (7)	146 (7)
O8W—H8B···O10W^x^	0.80 (7)	2.05 (8)	2.840 (8)	167 (12)
O8W—H8A···O17T^xi^	0.76 (7)	2.27 (10)	2.867 (9)	136 (12)
O9W—H9B···O12B^iii^	0.73 (8)	2.09 (10)	2.752 (8)	150 (14)
O9W—H9A···O6W^iii^	0.75 (8)	2.32 (11)	2.937 (10)	141 (13)
O10W—H10B···O7W^iii^	0.90 (7)	1.99 (7)	2.835 (8)	155 (9)
O10W—H10A···O23T	0.85 (7)	2.02 (7)	2.782 (8)	149 (9)
O11W—H11B···O10B	0.77 (8)	2.18 (9)	2.880 (8)	153 (14)
O11W—H11A···O18T^xii^	0.84 (7)	2.18 (8)	2.983 (8)	159 (12)

Symmetry codes: (iii) −*x*+3/2, −*y*+3/2, −*z*+1; (v) −*x*+1, −*y*+1, −*z*+1; (ix) *x*, −*y*+1, *z*−1/2; (vii) *x*−1/2, *y*+1/2, *z*; (vi) −*x*+3/2, *y*+1/2, −*z*+3/2; (i) −*x*+1, *y*, −*z*+1/2; (x) −*x*+3/2, *y*−1/2, −*z*+3/2; (xi) −*x*+3/2, −*y*+1/2, −*z*+1; (xii) −*x*+2, −*y*+1, −*z*+1.
